# Supplementary material for: Knowledge, attitudes and practices regarding bovine tuberculosis in cattle and humans in Malawi
Source: PLoS One. 2026 Feb 10;21(2):e0341968. doi: 10.1371/journal.pone.0341968 (PMC12890104; doi:10.1371/journal.pone.0341968)
Supplement: S1 Fig — (DOCX) [file pone.0341968.s001.docx]

**
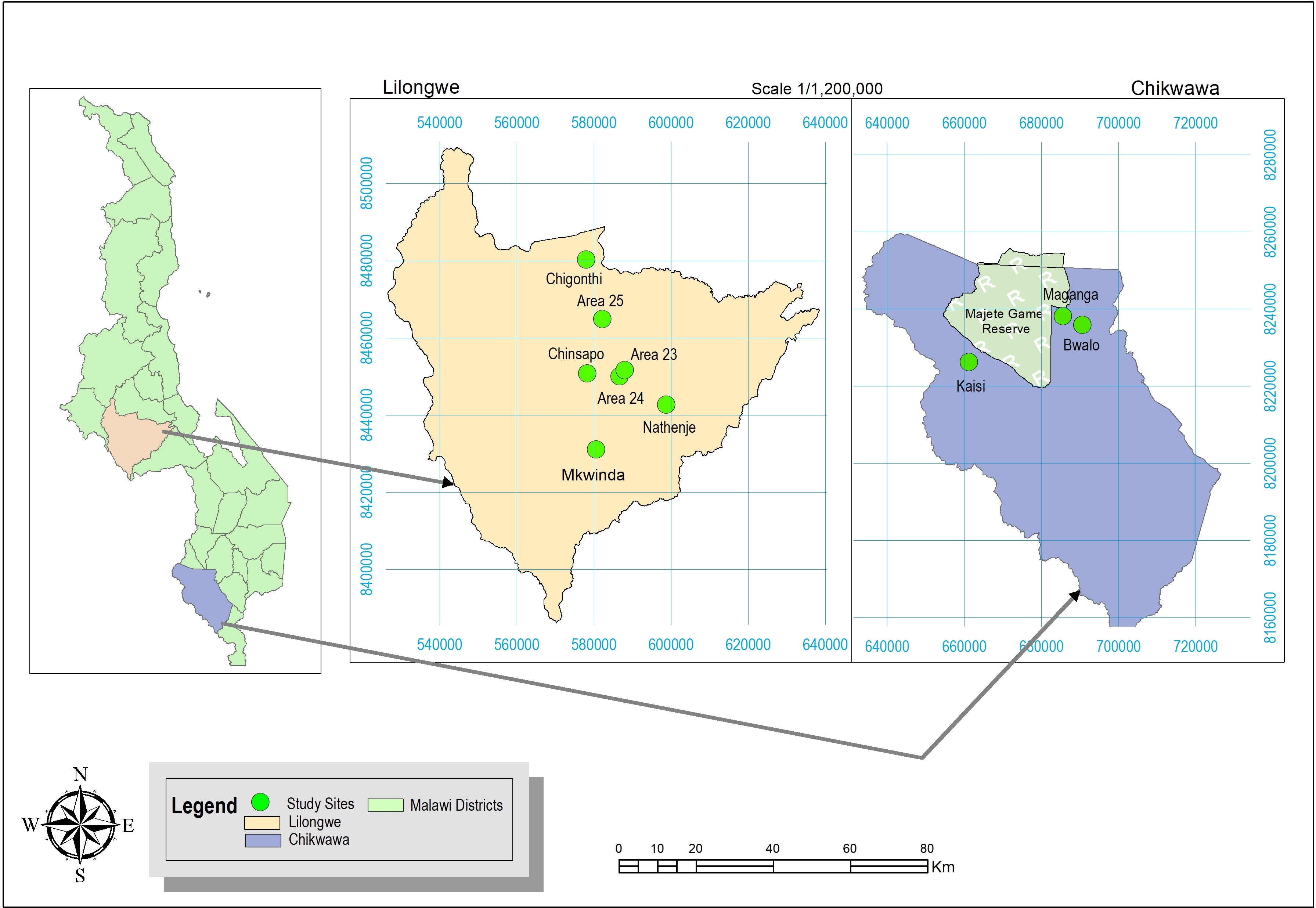
**

**S1 Fig.** **Study areas of knowledge, attitudes and practices about BTB (Source of shapefiles: NSO/Malawi and OCHA Field Information Services Section (**[**https://data.humdata.org/dataset/cod-ab-mwi**](https://data.humdata.org/dataset/cod-ab-mwi)**)).**
