# Supplementary figures and images for: Knowledge, attitudes and practices regarding bovine tuberculosis in cattle and humans in Malawi
Source: PLoS One. 2026 Feb 10;21(2):e0341968. doi: 10.1371/journal.pone.0341968 (PMC12890104; doi:10.1371/journal.pone.0341968)

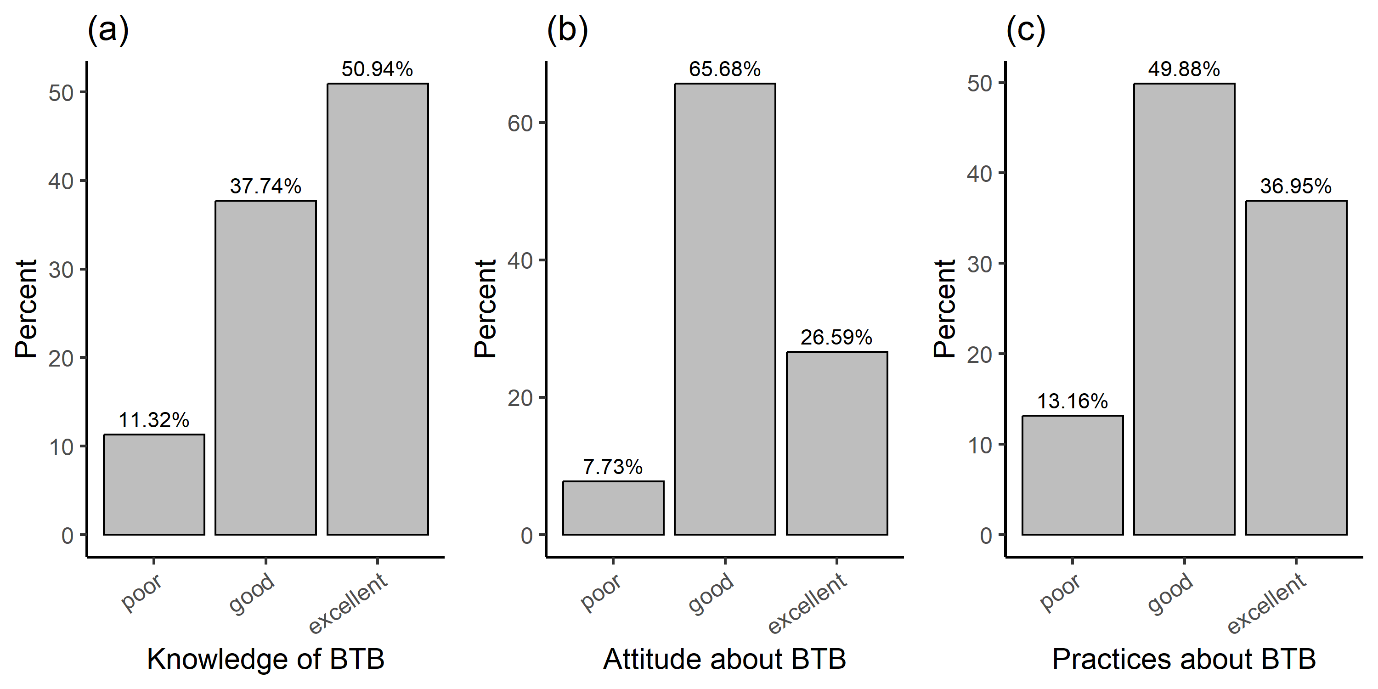


**S2 Fig.** **Distribution of knowledge, attitudes and practices about BTB.**

Supplement: S2 Fig — (DOCX) [file pone.0341968.s002.docx]

**S3 Appendix. Approval to collect data on knowledge, attitudes and practices about BTB.**


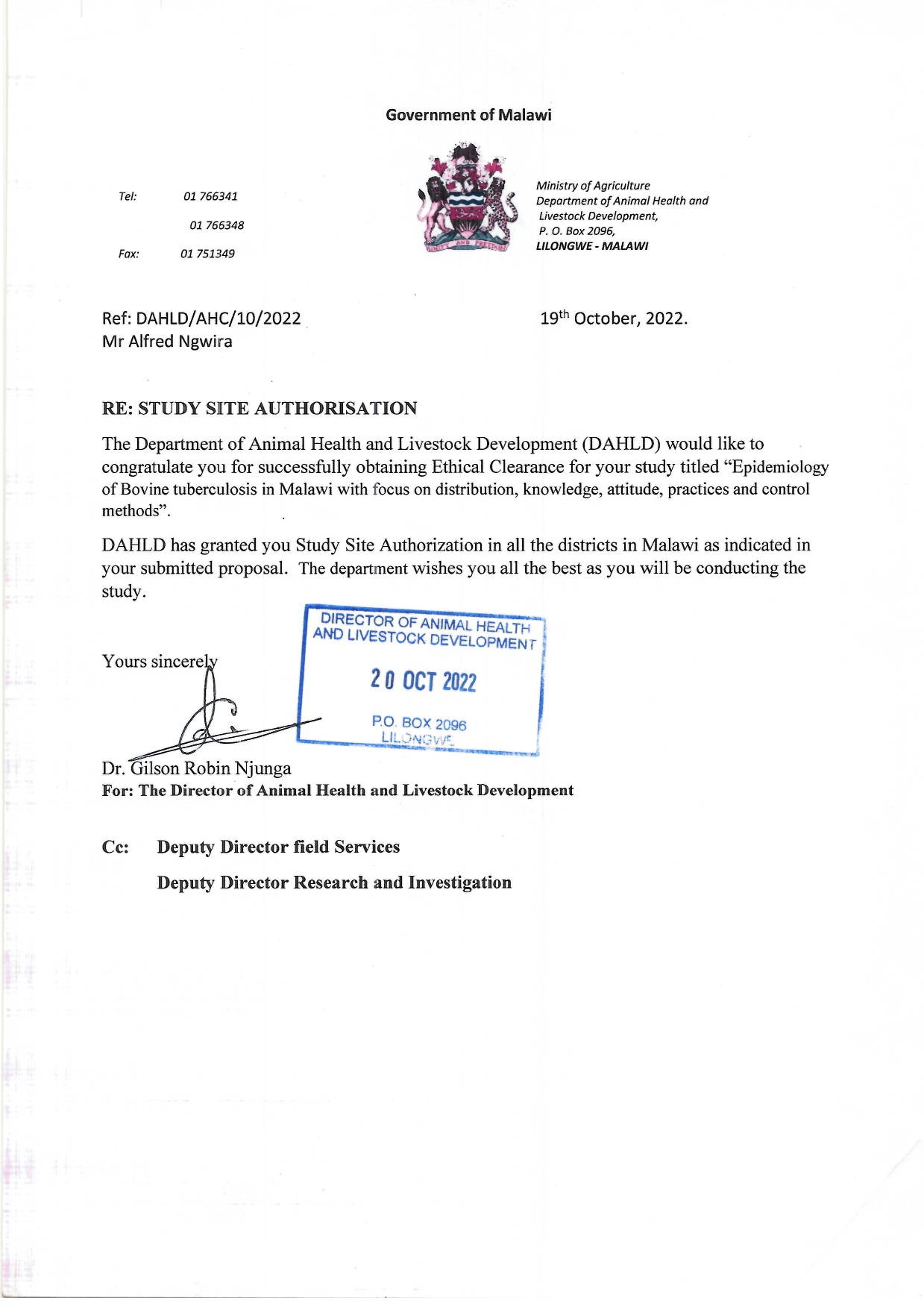

Supplement: S3 Appendix — (DOCX) [file pone.0341968.s010.docx]
