# Supplementary material for: Knowledge, attitudes and practices regarding bovine tuberculosis in cattle and humans in Malawi
Source: PLoS One. 2026 Feb 10;21(2):e0341968. doi: 10.1371/journal.pone.0341968 (PMC12890104; doi:10.1371/journal.pone.0341968)
Supplement: S1 Table — (DOCX) [file pone.0341968.s003.docx]

**S1 Table. Percentage of knowledge about BTB in cattle and humans.**

| **Question/Statement** | **Yes** | **No** | **Don’t know** |
| --- | --- | --- | --- |
| Heard of BTB that affect cattle | 85.71 | 14.06 | 0.23 |
| BTB is caused by microorganisms (bacteria) | 68.25 | 10.66 | 21.09 |
| BTB is not inherited from parents | 37.87 | 48.53 | 13.61 |
| BTB from cattle is communicable to humans | 74.09 | 17.05 | 8.86 |
| BTB from cattle is an airborne disease | 66.14 | 26.59 | 7.27 |
| Coughing is a sign of BTB | 57.82 | 39.23 | 2.95 |
| Losing weight is a sign of BTB | 47.50 | 49.09 | 3.41 |
| Low grade fever is a sign of BTB | 38.10 | 50.79 | 11.11 |
| Lymph enlargement is a sign of BTB | 38.27 | 43.05 | 18.68 |
| Cattle get BTB through proximity to wildlife or forest | 72.50 | 21.36 | 6.14 |
| Cattle get BTB by sharing water points with wildlife | 67.50 | 26.82 | 5.68 |
| Kraal with poor ventilation risk cattle infection | 84.81 | 13.15 | 2.04 |
| Intensive farming is a risk factor of BTB in cattle | 92.06 | 7.26 | 0.68 |
| Humans get BTB by eating or drinking raw meat and milk | 84.55 | 10.00 | 5.45 |
| Humans get BTB by sharing a house with livestock | 72.08 | 23.34 | 4.58 |
| Humans get BTB by sharing water points with cattle and wildlife | 68.26 | 28.54 | 3.20 |
| Humans get BTB through contact with infected livestock | 56.69 | 39.46 | 3.85 |
| Dairy farmers are at greater risk of getting infected with BTB | 74.38 | 22.45 | 3.17 |
| Meat handlers are at greater risk of contracting BTB | 72.56 | 24.49 | 2.95 |
| Boiling milk before drinking prevents BTB infection in humans | 87.05 | 11.59 | 1.36 |
| Cooking meat before eating help prevent BTB infection in humans | 80.27 | 18.14 | 1.59 |
| Biosecurity helps control BTB in cattle | 62.81 | 34.01 | 3.17 |
| Testing and slaughter help control BTB | 83.41 | 15.91 | 0.68 |
| Test and segregation help control BTB | 90.70 | 8.39 | 0.91 |
| Vaccination helps control BTB in humans and cattle | 95.69 | 3.40 | 0.91 |
| Education campaigns help control BTB | 93.88 | 5.44 | 0.68 |
