# Supplementary material for: Knowledge, attitudes and practices regarding bovine tuberculosis in cattle and humans in Malawi
Source: PLoS One. 2026 Feb 10;21(2):e0341968. doi: 10.1371/journal.pone.0341968 (PMC12890104; doi:10.1371/journal.pone.0341968)
Supplement: S2 Table — (DOCX) [file pone.0341968.s004.docx]

**S2 Table. Attitude about BTB in cattle and humans.**

| **Question/Statement** | **Strongly disagree** | **Disagree** | **Neutral** | **Agree** | **Strongly agree** |
| --- | --- | --- | --- | --- | --- |
| BTB is not a fatal disease | 66.67 | 13.38 | 8.62 | 3.63 | 7.71 |
| If humans have BTB then have HIV | 69.39 | 9.07 | 6.12 | 2.95 | 12.47 |
| Best treatment for BTB is from a witch doctor | 91.61 | 6.12 | 0.68 | 0.45 | 1.13 |
| When people go to hospital, they die of BTB | 70.29 | 17.69 | 6.12 | 2.27 | 3.63 |
| We should live and eat together with people or animals with BTB | 40.45 | 17.27 | 5.91 | 17.50 | 18.86 |
| I do not fear people or animals with BTB | 46.71 | 21.32 | 8.16 | 9.52 | 14.29 |
| Meat from infected animal can be eaten or sold | 81.18 | 7.03 | 1.81 | 7.03 | 2.95 |
| Milk from infected animal can be drunk or sold | 81.41 | 8.39 | 2.04 | 4.76 | 3.40 |
