# Supplementary material for: Knowledge, attitudes and practices regarding bovine tuberculosis in cattle and humans in Malawi
Source: PLoS One. 2026 Feb 10;21(2):e0341968. doi: 10.1371/journal.pone.0341968 (PMC12890104; doi:10.1371/journal.pone.0341968)
Supplement: S3 Table — (DOCX) [file pone.0341968.s005.docx]

**S3 Table. Practices about BTB in cattle and humans.**

| **Question/Statement** | **Yes** | **No** | **Don’t know** |
| --- | --- | --- | --- |
| Do you sometimes eat raw or undercooked meat? | 22.05 | 77.50 | 0.45 |
| Do you sometimes drink unboiled or raw milk? | 33.33 | 66.21 | 0.45 |
| Do you sometimes graze animals close to a wildlife reserve or forest? | 50.23 | 48.86 | 0.91 |
| Do you first buy medicine from a veterinary shop if your cattle are sick before calling for a veterinary doctor? | 33.33 | 66.21 | 0.45 |
| Do you go to the hospital late when you are sick? | 22.45 | 77.55 | 0.00 |
| Do you first go to the witch doctor if you are sick? | 3.19 | 96.81 | 0.00 |
| Do you first buy medicine from the pharmacy if you are sick? | 49.21 | 50.79 | 0.00 |
| Do you keep some livestock in your house? | 42.18 | 57.82 | 0.00 |
| Do your cattle mix with other people cattle during grazing? | 70.29 | 29.71 | 0.00 |
| Do you eat or sell meat from a sick animal? | 18.37 | 81.41 | 0.23 |
| Do you drink or sell milk from a sick animal? | 8.01 | 91.53 | 0.46 |
| Do you sometimes not use protective wear when handling animal products such as milk or meat? | 75.28 | 22.45 | 2.27 |
