# Supplementary material for: Knowledge, attitudes and practices regarding bovine tuberculosis in cattle and humans in Malawi
Source: PLoS One. 2026 Feb 10;21(2):e0341968. doi: 10.1371/journal.pone.0341968 (PMC12890104; doi:10.1371/journal.pone.0341968)
Supplement: S4 Table — (DOCX) [file pone.0341968.s006.docx]

**S4 Table. Comparison of standard errors (SE) and coefficients.**

| **Variable** | **Mean difference** | **Estimate** | **Standard error** | **95% Confidence interval** |
| --- | --- | --- | --- | --- |
| SE of effects of knowledge | Univariate-Multivariate | -0.024 | 0.337 | (-0.712, 0.663) |
| SE of effects of attitude | Univariate-Multivariate | -0.040 | 0.309 | (-0.669, 0.589) |
| SE of effects of practice | Univariate-Multivariate | -0.017 | 0.136 | (-0.293, 0.258) |
| Coefficient of age (18–30) | Attitude-Practice | 4.218 | 3.111 | (-1.899, 10.334) |
| Coefficient of age (31–45) | Attitude-Practice | 3.085 | 3.139 | (-3.088, 9.258) |
| Coefficient of secondary education | Attitude-Practice | -2.066 | 1.028 | (-4.088, -0.044) |
| Coefficient of higher education | Attitude-Practice | -3.536 | 1.598 | (-6.677, -0.395) |
| Coefficient of student | Attitude-Practice | 1.442 | 2.967 | (-4.391, 7.274) |
