# Supplementary material for: Knowledge, attitudes and practices regarding bovine tuberculosis in cattle and humans in Malawi
Source: PLoS One. 2026 Feb 10;21(2):e0341968. doi: 10.1371/journal.pone.0341968 (PMC12890104; doi:10.1371/journal.pone.0341968)
Supplement: S5 Table — (DOCX) [file pone.0341968.s007.docx]

**S5 Table. Residual correlation and *p*-values.**

| **Pearson correlation coefficients** | | | |
| --- | --- | --- | --- |
|  | knowledge | attitude | practice |
| knowledge | 1 |  |  |
| attitude | -0.140 | 1 |  |
| practice | -0.021 | 0.218 | 1 |
| ***p*-values** | | | |
|  | knowledge | attitude | practice |
| knowledge | - |  |  |
| attitude | 0.004 | - |  |
| practice | 0.675 | 0.000 | - |
