# Supplementary material for: Knowledge, attitudes and practices regarding bovine tuberculosis in cattle and humans in Malawi
Source: PLoS One. 2026 Feb 10;21(2):e0341968. doi: 10.1371/journal.pone.0341968 (PMC12890104; doi:10.1371/journal.pone.0341968)
Supplement: S1 Appendix — (DOCX) [file pone.0341968.s008.docx]

**S1 Appendix. English questionnaire on knowledge, attitude and practices about BTB in cattle and humans in Malawi**

***Introduction***

I am Alfred Ngwira, a PhD student of Sokoine University of Agriculture conducting research on knowledge, attitude and practices about bovine tuberculosis in Malawi. I am requesting for your consent to participate in this study. Your agreement to participate is highly appreciated. Do you agree to participate in this research? Yes , No .

***Section A: Background characteristics***

Age in years:

Sex (male/female):

Education level (no education/primary/secondary/higher):

Occupation: Farmer , Student , Business , Employee , Labour

Residence (urban/rural):

Marital status: Married , Not married

Income: Low , Middle , High

***Section B: Knowledge about BTB in cattle and humans***

| **QN** | **Question/Statement** | **Yes** | **No** | **Don’t know** |
| --- | --- | --- | --- | --- |
| B1 | Heard of BTB that affect cattle |  |  |  |
| B2 | BTB is caused by microorganisms (bacteria) |  |  |  |
| B3 | BTB is not inherited from parents |  |  |  |
| B4 | BTB from cattle is communicable to humans |  |  |  |
| B5 | BTB from cattle is an airborne disease |  |  |  |
| B6 | Coughing is a sign of BTB |  |  |  |
| B7 | Losing weight is a sign of BTB |  |  |  |
| B8 | Low grade fever is a sign of BTB |  |  |  |
| B9 | Lymph enlargement is a sign of BTB |  |  |  |
| B10 | Cattle get BTB through proximity to wildlife or forest |  |  |  |
| B11 | Cattle get BTB by sharing water points with wildlife |  |  |  |
| B12 | Kraal with poor ventilation risk cattle infection |  |  |  |
| B13 | Intensive farming is a risk factor of BTB in cattle |  |  |  |
| B14 | Humans get BTB by eating or drinking raw meat and milk |  |  |  |
| B15 | Humans get BTB by sharing a house with livestock |  |  |  |
| B16 | Humans get BTB by sharing water points with cattle and wildlife |  |  |  |
| B17 | Humans get BTB through contact with infected livestock |  |  |  |
| B18 | Dairy farmers are at greater risk of getting infected with BTB |  |  |  |
| B19 | Meat handlers are at greater risk of contracting BTB |  |  |  |
| B20 | Boiling milk before drinking prevents BTB infection in humans |  |  |  |
| B21 | Cooking meat before eating help prevent BTB infection in humans |  |  |  |
| B22 | Biosecurity helps control BTB in cattle |  |  |  |
| B23 | Testing and slaughter help control BTB |  |  |  |
| B24 | Test and segregation help control BTB |  |  |  |
| B25 | Vaccination helps control BTB in humans and cattle |  |  |  |
| B26 | Education campaigns help control BTB |  |  |  |

***Section C: Attitude or perception about BTB in cattle and humans***

| **QN** | **Question/Statement** | **Strongly disagree** | **Disagree** | **Neutral** | **Agree** | **Strongly Agree** |
| --- | --- | --- | --- | --- | --- | --- |
| C1 | BTB is not a fatal disease |  |  |  |  |  |
| C2 | If humans have BTB then have HIV |  |  |  |  |  |
| C3 | Best treatment for BTB is from a witch doctor |  |  |  |  |  |
| C4 | When people go to hospital, they die of BTB |  |  |  |  |  |
| C5 | We should live and eat together with people or animals with BTB |  |  |  |  |  |
| C6 | I do not fear people or animals with BTB |  |  |  |  |  |
| C7 | Meat from infected animal can be eaten or sold |  |  |  |  |  |
| C8 | Milk from infected animal can be drunk or sold |  |  |  |  |  |

***Section D: Practices about BTB in cattle and humans***

| **QN** | **Question/Statement** | **Yes** | **No** | **Don’t know** |
| --- | --- | --- | --- | --- |
| D1 | Do you sometimes eat raw or undercooked meat? |  |  |  |
| D2 | Do you sometimes drink unboiled or raw milk? |  |  |  |
| D3 | Do you sometimes graze animals close to a wildlife reserve or forest? |  |  |  |
| D4 | Do you first buy medicine from a veterinary shop if your cattle are sick before calling for a veterinary doctor? |  |  |  |
| D5 | Do you go to the hospital late when you are sick? |  |  |  |
| D6 | Do you first go to the witch doctor if you are sick? |  |  |  |
| D7 | Do you first buy medicine from the pharmacy if you are sick? |  |  |  |
| D8 | Do you keep some livestock in your house? |  |  |  |
| D9 | Do your cattle mix with other people cattle during grazing? |  |  |  |
| D10 | Do you eat or sell meat from a sick animal? |  |  |  |
| D11 | Do you drink or sell milk from a sick animal? |  |  |  |
| D12 | Do you sometimes not use protective wear when handling animal products such as milk or meat? |  |  |  |
