# Supplementary material for: Knowledge, attitudes and practices regarding bovine tuberculosis in cattle and humans in Malawi
Source: PLoS One. 2026 Feb 10;21(2):e0341968. doi: 10.1371/journal.pone.0341968 (PMC12890104; doi:10.1371/journal.pone.0341968)
Supplement: S2 Appendix — (DOCX) [file pone.0341968.s009.docx]

**S2 Appendix. Chichewa questionnaire on knowledge, attitude and practices about BTB in cattle and humans in Malawi**

Chizindikiro cha wonfunsidwa

**KUZIWA, MAGANIZIDWE NDI ZOCHITA ZOKHUZANA NDI MATENDA A TB YOZERA KU NG’OMBE MU MALAWI.**

**Uthenga wofunikira**

Chizindikiro cha wofunsa:

Makina a inteneti a malo ofunsira**:**

**Mau oyamba**

Mulibwanji? Ine ndine Alfred Ngwira, wophunzira wa za ukachechede pa sukulu ya Sokoine ya zamalimidwe muziko la Tanzania. Ndikuchita kafukufuku wa matenda a TB yodzera ku ng’ombe mu Malawi makamaka, kudziwa, kaganizidwe ndi kachitidwe ka anthu paza matendawa. Amene atenge nawo mbali ndi okhawo amene ali omasuka kutero. Ndikuthokoza kwambiri chifukwa chovomera kutenga nawo mbali. Khalani omasuka.

**Gawo A: Uthenga woyamba wa munthu**

Zaka:

Chibadidwe: Muna , Kazi

Sukulu: Sinapite kusukulu , Pulaimale , Sekondale , Ukachechede

Ntchito: Mlimi ,Wophunzira , Bisimisi , Yolembedwa , Ganyu

Malo okhala: Mtauni , Kumudzi

Kukwatiwa: Wokwatira , Wosakwatira

Kupeza ndalama pamwezi: 0 – 100 MK , > 100 - 500 MK , > 500 MK

**Gawo B: Kuziwa za matenda a TB wozera kung’ombe**

| **QN** | **Ndemanga/Funso** | **Eya** | **Ayi** | **Sindidziwa** |
| --- | --- | --- | --- | --- |
| B1 | Munamvapo za matenda a TB wogwira ng**’**ombe? |  |  |  |
| B2 | TB yogwira ng**’**ombe imadza ndi tizilimbo (ta bakiteliya)? |  |  |  |
| B3 | TB yodzera ku ng’ombe simatengedwa pobadwa? |  |  |  |
| B4 | Kodi TB yogwira ng**’**ombe imathaso kugwira anthu? |  |  |  |
| B5 | Kodi TB ya ng**’**ombe imafalisidwa kudzera popuma mumpweya? |  |  |  |
| B6 | Kodi kukhosomola ndi chizindikiro cha TB ku ng**’**ombe ndi anthu? |  |  |  |
| B7 | Kodi kuwonda thupi ndi chizindikiro cha TB ku ng**’**ombe ndi anthu? |  |  |  |
| B8 | Kodi kumva mphepo ndi chizindikiro cha TB ku ng**’**ombe nd anthu? |  |  |  |
| B9 | Kodi kutupa mitsempha (***lymph nodes***) ndichizindikiro cha TB ku ng**’**ombe ndi anthu? |  |  |  |
| B10 | Kodi ng**’**ombe zokhala pafupi ndi nyama zamutchire ndi thengo zimakhala pachiophyezo kutenga TB? |  |  |  |
| B11 | Kodi ng**’**ombe zimatenga TB pokumwa madzi limozi ndi nyama zamutchire? |  |  |  |
| B12 | Kodi khola lopanda mpweya wokwanira limaopyeza ng**’**ombe kutenga TB? |  |  |  |
| B13 | Kodi kuweta/kusunga ng**’**ombe mothinana kumaophyeza ng**’**ombe kutenga TB? |  |  |  |
| B14 | Kodi anthu amatenga TB pokumwa mkaka kapena kudya nyama yaiwisi? |  |  |  |
| B15 | Kodi anthu amatha kutenga TB ya ng**’**ombe pokhala/kugona limozi ndi ngo’mbe munyumba? |  |  |  |
| B16 | Kodi anthu amatha kutenga TB ya ng**’**ombe pomwera limozi madzi ndi ng**’**ombe ndi nyama za mutchire? |  |  |  |
| B17 | Kodi anthu amatenga TB pogundana/pogwira nyama yamatenda a TB? |  |  |  |
| B18 | Kodi alimi a ng**’**ombe za mkaka ali pachiophyezo chachikulu chotenga TB? |  |  |  |
| B19 | Kodi opha ndi ogulisa nyama ali pachiophyezo chotenga TB yozera kung’ombe? |  |  |  |
| B20 | Kodi kuphika mkaka kumachepesa chiophyezo cha TB yozera kung’ombe ku anthu? |  |  |  |
| B21 | Kodi kuphika nyama kumateteza anthu ku matenda a TB yozera kung’ombe? |  |  |  |
| B22 | Kodi kumangira mpanda khola la ngo’mbe kumateteza ng**’**ombe ku matenda a TB? |  |  |  |
| B23 | Kodi kuyeza ndi kupha ng**’**ombe zopezeka ndi matenda a TB kumachepesa matenda a TB? |  |  |  |
| B24 | Kodi kuyeza ndi ku patula ng**’**ombe zopezeka ndi matenda a TB kumachepesa matenda a TB? |  |  |  |
| B25 | Kodi katemela amathandiza kuchepesa kufala kwa TB mu ng**’**ombe ndi anthu? |  |  |  |
| B26 | Kodi kupereka maphunziro/uphungu kwa anthu kumachepesa kufala kwa TB ku ng**’**ombe ndi anthu? |  |  |  |

**Gawo C: Maganizidwe a anthu pa TB ya ng’ombe**

| **QN** | **Ndemanga/Funso** | **Ndikukana kwathunthu** | **Ndikukana** | **Pakatikati**  **(Sindikuziwa)** | **Ndikuvomereza** | **Ndikuvomereza kwathunthu** |
| --- | --- | --- | --- | --- | --- | --- |
| C1 | TB yodzera ku ng**’**ombe simapha ng**’**ombe kapena anthu |  |  |  |  |  |
| C2 | Anthu akakhala ndi TB ndiye kuti ali ndi Edzi (HIV) |  |  |  |  |  |
| C3 | Kuchira TB kwabwino ndi kwa a sing**’**anga |  |  |  |  |  |
| C4 | Anthu akapita ku chipatala ndi TB amafera konko, sabwerako amoyo. |  |  |  |  |  |
| C5 | Tithatha kukhala ndi kumwa limozi ndi anthu kapena ziweto zokhala ndi TB |  |  |  |  |  |
| C6 | Sindimaopa anthu ndi ng’ombe zokhala ndi TB |  |  |  |  |  |
| C7 | Nyama yochokera ku ng**’**ombe ya TB itha kudyedwa ndi kugulisidwa |  |  |  |  |  |
| C8 | Mkaka wochokera ku ng**’**ombe za matenda titha kumwa kapena kugulisa |  |  |  |  |  |

**Gawo D: Zochita zokhuzana ndi TB yozera ku ng’ombe**

| **QN** | **Ndemanga/Funso** | **Eya** | **Ayi** | **Don’t know** |
| --- | --- | --- | --- | --- |
| D1 | Kodi nthawi zina mumadya nyama yaiwisi kapena yosaphikisa? |  |  |  |
| D2 | Kodi nthawi zina mumamwa mkaka wosaphika kapena wawuwisi? |  |  |  |
| D3 | Kodi nthawi zina mumadyesa ziweto kufupi ndi nyama za mutchire kapena kuthengo? |  |  |  |
| D4 | Kod nthawi zina mumagula kaye makhwala a ziweto zikadwala musanaitane alangizi a ziweto? |  |  |  |
| D5 | Kodi mumapita kuchipatala mochedwa mukadwala? |  |  |  |
| D6 | Kodi mumapita kaye kwa a sing**’**anga musanapite kuchipatala mukadwala? |  |  |  |
| D7 | Kodi mumagula kaye mankhwala ku sitolo musanapite kuchipatala? |  |  |  |
| D8 | Kodi nthawi zina mumasunga ziweto zina monga mbuzi munyumba? |  |  |  |
| D9 | Kodi nthawi zina ziweto zanu zimaphatikizana ndiza anthu ena mukamadyesa? |  |  |  |
| D10 | Kodi nthawi zina mumadya ndi kugulitsa nyama yoti yafa yokha ndimatenda? |  |  |  |
| D11 | Kodi nthawi zina mumamwa ndi kugulisa mkaka kuchokera kunyama yodwala? |  |  |  |
| D12 | Kodi nthawi zose mumagwiritsa ntchito zovala zozitetedzera monga magluvosi mukamagwira nyama kapena mkaka wa ng’ombe? |  |  |  |
