# Supplementary material for: Knowledge, attitudes and practices regarding bovine tuberculosis in cattle and humans in Malawi
Source: PLoS One. 2026 Feb 10;21(2):e0341968. doi: 10.1371/journal.pone.0341968 (PMC12890104; doi:10.1371/journal.pone.0341968)
Supplement: Attachment — Submitted filename: Response to reviewers.docx [file pone.0341968.s012.docx]

**RESPONSE TO REVIEWERS (*Author responses are in italics*)**

Reviewer #1: A KAP survey should assess knowledge, attitudes, and practices directly related to bovine TB. The included variables such as age, sex, education, occupation, marital status, and income are purely demographic and socioeconomic, lacking disease-specific insights. Without assessing awareness, risk perception, and preventive behaviours, the study fails to provide actionable findings for bovine TB control and intervention.

***Response:*** *The disease specific questions are the in supplementary file in the questionnaire (S1 Appendix) and their results are in supplementary tables S1-S3 Tables. We suspect the reviewer did not check the supplementary information. The mentioned demographic characteristics were used to determine factors of KAP. This is a common practice when collecting KAP data where disease specific questions plus demographic characteristics are collected with the aim of determining their influence on KAP. See e.g Hamid et al (2024)* *www.onehealthjournal.org/Vol.10/No.1/11.pdf**. Nevertheless, the paper has been updated in the “Abstract”, “’Results” and “discussion”’ sections detailing findings on disease specific questions.*

Reviewer #2: - This paper would add critical information regarding farmers and cattle handlers’ knowledge and practices with respect to bovine tuberculosis in Malawi. As it stands, the paper lacks information regarding the various findings obtained from the survey instrument, the survey development and recruitment processes used, discussion of the findings in relation to similar studies in literature, and the implications of this research. I believe addition of these important pieces of information will strengthen the manuscript. I have highlighted areas where additions should be made below.

- Consider adding more information in the abstract: for instance, information regarding the questionnaire used in the methods, along with other important findings obtained in the results sub-section.

***Response:*** *We have added the extra result information in the abstract. Page 1, line 29-34.*

- In the abstract, in the sentence ‘Overall knowledge, attitude, and practices about BTB among participants were good’ – how do you define ‘good’?

***Response:*** *We added the percentage statistics to show how good it was. Page 1, line 29-34.*

- In the keywords section, consider adding the keywords ‘KAP survey’ or ‘knowledge, attitude, practice study’.

***Response:*** *We have added the key word “KAP survey”. Page 2, line 2,3.*

- The introduction section could do better with additional information and more context on bovine TB. What is the burden of BTB in Malawi as compared to its neighboring countries? How does bovine TB differ from zoonotic TB? Are symptoms similar between bovine and human TB? How does bovine TB occur? What is your hypothesis regarding this particular study population on bovine TB?

***Response:*** *We have added information about BTB burden in Malawi and how is related to neighbours. Page 2, line 7-9.*

- The questionnaire used for this study- was it developed for this study, or has it been adapted from other studies? If so, please provide citations. Were the surveys conducted in person or via mail? Please provide more information on the recruitment and study advertisement processes used. Did you provide any incentives to participants as part of the study? What was the language in which the KAP survey was administered in? Were translation and back-translation processes needed for the survey?
- Which software/platform did you use to perform the statistical analyses?

***Response:*** *We have added all the requested information about the survey methodology on page 3-4. i.e*

1) *Citation of where we adapted question items for the questionnaire is indicated on Page 3, line 35, 36.*
2) *Whether survey was in person/email is indicated on Page 4, line 4, 5.*
3) *Provision of incentives is indicated on Page 4, line 5.*
4) *Language used in the questionnaire is indicated on page 4, line 5,6.*
5) *Software used is indicated on page 4, line 32,33.*

- I am curious, is there any reason as to why you chose to show that higher scores for knowledge corresponds with more knowledge whereas higher scores in attitudes and practices corresponds to low perceptions and practices?

***Response:*** *It was not by choice, but by* *the measurement scales of specific items for knowledge (0,1), attitude (1,2,3,4,5) and practices (0,1). In this regard, a one for knowledge meant correct knowledge and a zero corresponded to wrong or don’t know. A lower value on the attitude Likert scale (e.g 1,2) corresponded to negative or good attitude about risky behaviours, and a higher value e.g 4, 5, indicated positive or bad attitude about risky behaviours. Similarly, a higher practice score i.e a 1, meant malpractice, and a lower practice score i.e a 0, meant no malpractice or good practice.*

- I think it would be helpful and informative if you added separate findings obtained for the knowledge, attitude, and practice sections of the survey instrument in the results section, in addition to what is currently presented.

***Response:*** *I added the extra information in the results section. See Page 4, line 36 to page 5 line 17.*

- How do the results from this KAP study stand in relation to other KAP studies on human/bovine TB? Are these results similar or different?

***Response:*** *The information has been added in the discussion. E.g Page* *11 line 5-17.*

- Please discuss in more detail the interpretation of the results and regression analyses and the implications of these findings in context with other studies on this topic. Was the KAP survey successful in capturing knowledge gaps and data gaps among the surveyed participants in Malawi?

***Response****: Extra details were added in Discussion e.g on regression on Page 11, line 28-39.*

- What would be some future directions as an outcome of this study?

***Response:*** *Future directions are indicated in the conclusion section.*
