# Supplementary material for: Knowledge, attitudes and practices regarding bovine tuberculosis in cattle and humans in Malawi
Source: PLoS One. 2026 Feb 10;21(2):e0341968. doi: 10.1371/journal.pone.0341968 (PMC12890104; doi:10.1371/journal.pone.0341968)
Supplement: Attachment — Submitted filename: Response_to_reviewers_auresp_2.docx [file pone.0341968.s013.docx]

**Response to reviewers**

Reviewer #3:

Comment to the Editor
I found the paper interesting, and useful. I recommend acceptance and addressing my quite minor issues.
General Comments:
The manuscript by Ngwira et al. provides some interesting and useful results regarding the knowledge, attitudes, and practices (KAP) regarding BTB in high-burden areas of Malawi.
This study demonstrates how demographics influence KAP jointly. Insights gained from this KAP assessment can inform public health officials for targeted health campaigns to reduce the spread of BTB.
Overall, the paper was well structured. There are some points that should be addressed, as listed below.
Specific Comments:
Page 4, Line 2 – 4, please explain how income level was determined.

***Response:*** *Participants' income was measured through their responses to the amount of money they could approximately make from different economic activities in a month. This statement has been added on page 3, line 45-46.*

Page 4, Line 20: Add “≥” before 50%

***Response:*** *We thank reviewer for this suggestion. Basically, the first category is <50%****,*** *the second is 50 to < 75% and third is ≥75, which means by writing 2nd category 50 to < 75% we are already including 50, therefore ≥ before 50 may not be necessary.*

Page 5, Line 7 – 9: If the statement implies statistical significance, please add the corresponding p-values (or confidence intervals). If no statistical test was performed, avoid using “significant” and instead use neutral phrasing such as: “A considerable proportion of participants…” or “A high proportion of participants...”

***Response:*** *We tried to use words like “considerable”, “substantial”, or “notable”, throughout the manuscript where there was “significant” e.g See page 5, line 4 and 5.*

Page 5, Line 29: Mention which test was used to test significant differences in standard errors and coefficients.

***Response:*** *We have mentioned the test statistic that it was t-test on page 4 line 28.*

Page 5, Line 39-43: If the values in table 5 are Pearson correlations among the KAP scores, the correct notation is r.

***Response:*** *We have modified by using r instead of rho,* $\rho$*. See page 5, line 32-35.*

Table 1 and 2: For both the univariate and multivariate regression tables, please include p-values for each coefficient to indicate statistical significance.

***Response:*** *We thank reviewer for this suggestion. Basically, the significance of the coefficients is also determined by their 95% confidence intervals, i.e if the interval includes zero, it is considered as not significant and if the interval excludes zero, it is significant. Therefore, we still think it would suffice to have intervals only, otherwise including the p-values, we feel it will seem duplicating and overcrowding the tables. Moreover, just using the coefficient and its 95% CI is not new, see e.g Table 5 in Gebremeskel et al (2022) at* [*https://pdfs.semanticscholar.org/a122/ca24e4f586a174847a3c78a472e418f1131a.pdf*](https://pdfs.semanticscholar.org/a122/ca24e4f586a174847a3c78a472e418f1131a.pdf%20) *and Table 4 and 5 in Worku et al (2025) at* [*https://www.mdpi.com/2072-6643/17/2/252*](https://www.mdpi.com/2072-6643/17/2/252).

Table 3: Please clarify this table. Please include p-values for each coefficient to indicate statistical significance.

***Response:*** *Table 3 has been moved to supplementary materials as table S4 Table according to suggestion by co-author Prof. Samuel Manda.* *We have clarified what is in Table 3 (now S4 Table) on page 4, line 28-30. Similarly, as in the previous response, we feel to maintain only coefficients and their 95% confidence intervals without p-values to avoid overcrowding. Significance of coefficients is determined by intervals.*

S1 Appendix Question
In Background characteristics, how was income determined?

***Response:*** *Participants' income was measured through their responses to the amount of money they could approximately make from different economic activities in a month. This statement has been added on page 3, line 45-46.*

S1 Appendix Question/Statement 3 “BTB is not inherited from parents”:
The term hereditary is not appropriate because BTB is not a genetic disease; it implies a genetic predisposition, which is incorrect. Therefore, I understand that answering “Yes” would reflect correct knowledge. However, I wonder if the statement was intended to address vertical transmission instead. The translation from the African language (based on Google) reads: “Bovine TB is not acquired at birth.” If this is the intended interpretation, then “No” would be correct, since BTB can be transmitted vertically via milk or in utero.
To ensure accuracy and consistency in the manuscript, I recommend reviewing the survey translation carefully. Also, ensure consistency between the questions shown in S1 Table 1 and those in the S1 Appendix.

***Response:*** *The accuracy and consistency of question items in S1 Table 1 and those in the S1 Appendix was reviewed and validated.*

If the correct explanation is the former then I suggest rephrasing the results section for this question to “For Among participants, 37.87% answered ‘Yes,’ correctly recognizing that BTB is not transmitted through heredity.”

***Response:*** *The former is correct and is line with what we had initially written, nevertheless the statement has been slightly rephrased as advised by the reviewer. See page 4, line 44-46.*

S1 Appendix, Section B
Knowledge about BTB in cattle and humans: Are questions B7, B8, B9 specific for BTB in humans or cattle? Or is both?

***Response:*** *We were assuming they are for both since clinical signs of BTB are indistinguishable in both cattle and humans. See Khairullah et al (2024) at* [*https://pubmed.ncbi.nlm.nih.gov/39055751*](https://pubmed.ncbi.nlm.nih.gov/39055751)

Please clarify in page 5 line 1 and 2 “Participants also demonstrated limited knowledge on clinical signs, such as lymph node enlargement, weight loss, and low-grade fever.”

***Response:*** *This information is based on percentages distribution in table S1 Table. In text citation of table S1 Table has been made at the beginning of the paragraph, and our understanding is that readers would be referring to this table upon reading this information. We avoid including percentages to avoid overcrowding.*
